# Cases from Practice

**Published:** 1881-12

**Authors:** D. C. McLaren

**Affiliations:** Montreal


### CASES FROM PRACTICE.

D. C. McLaren, M. D., Montreal.

April 8th, 1881.

*Bronchitis.*—Miss B. æt. 50, has been suffering for six weeks with Bronchitis under allopathic treatment. Thought by herself and friends to be dying. So weak cannot sit up in bed. Entire loss of appetite. Hectic at various times, both day and night. Great oppression of breathing across upper part of chest. Slight cough. Breathing very shallow—large rales can be heard several feet away. Expectorates large quantities of thick green mucus, which seems to accumulate in trachea and comes up without effort. Pulse too quick and shabby to be counted. Began treatment with a dose of *Nux vomica* 200 to antidote previous cough mixtures.

9th. On awaking this morning was seized with faintness and great prostration. Breathing very labored. Eyes glazed and staring. *Stannum* 2c (J.), one dose.

10th. Expectoration much diminished—breathing a little easier. Pulse 90. No medicine.

11th. Another attack of prostration on awaking, worse than before. *Stannum* 2c (J.), one dose.

12th. Improving. No medicine.

13th. Short, sharp cough. Headache and flushed cheeks. *Bell.* 60M, one dose.

14th. Improving. No medicine.

15th. Improving. *Hepar* 500, one dose.

16th. Improving. No medicine.

17th. Convalescent. *Hepar* 1000.

25th. Slight cough after exertion. *Hepar* 1000.

27th. Has been up for several days. Now working as usual about the house.

*Diphtheria.*—Miss M. L., aet. 14.

Sept. 12th, 1881. Not feeling very well for past week. At 11 A. M., in school had a severe rigor, came home blue and cold. Quickly followed by fever, sore throat and headache.

5.30 P. M. Pulse 160, temp. 105°. Face red and flushed, pupils dilated. Tongue pinky white with prominent red papillæ. No appetite. Throat painfully sore on right side only. *Bell.* 60M, one dose.

13th, 10 A. M. Had a very restless night with heavy breathing. No appetite. Headache gone and throat not so sore. Right side of neck considerably swollen. Mouth very dry. Tongue red and hot; white posteriorly. Patch of membrane on right tonsil, size of shilling, commencing on left tonsil. No motion of bowels since 11th. Pulse 140.

13th, 5 P. M. Continued improvement. Pulse 120. Temperature 103$\frac{4}{5}$. No sore throat. Swallows well and appetite fairly good. Profuse sweat came on this afternoon and lasted all night. Watery coryza. Membrane equal on both sides. No medicine to-day.

14th, 10 A. M. Pulse 130. Temp. 100$\frac{4}{5}$. Continued improvement. Slept well. Perspired all night. Membrane loosening on left side; right is still solid. Mucus membrane less angry and tumefied. Coryza less.

14th, 5 P. M. Pulse 120. Temp. 101°. Has been coughing up pieces of membrane which on inspection find to have come from left side, as right is still firm. Feels well and cheerful. No soreness of throat or pain on swallowing. No motion of bowels yet. No medicine.

Sept. 15th, 10 A. M. Pulse 120. Temp. 102°. Coryza less. Membrane nearly all gone from left side. A good deal of heat of skin. One dose *Bell.*, 60M. Bowels moved naturally during the day.

Sept. 16th. Pulse 110. Temp. 99°. Membrane gone from left side and loosening on right. No medicine.

Sept. 17th. Pulse 100. Temp. 98° Membrane almost entirely gone from both sides. No medicine.

Sept. 18. Pulse 80. Temp. 98°. No sign of membrane at all; slight redness of tonsils. Tongue normal pink. Lips dry. Profuse epistaxis yesterday afternoon and again during the night. Dismissed the case with a dose of *Bryonia* 2c (J.), to be taken at bed-time.

[The points of interest in this case are the marked direction of the disease, and its retrogression of cure—the critical sweat of second day—and a secondary crisis (?) by epistaxis on the sixth day.]

*Orchitis.*—A. F. P. 29.

Sept. 15th, 1881. An allopathic physician, has been suffering more or less for last fortnight with swollen left testicle. Yesterday pain grew so severe he almost fainted—took ½ gr. of morphia without any relief. To-day cannot walk on account of the excruciating pain when anything touched it; "*sore as a boil*, if not worse," he says. Has read Bumstead on venereal diseases, and wants to know if I am giving him *Pulsatilla?* Prescribed *Hepar* 1000, one dose in water, to be taken several times during the hour and then discontinued. In two hours was able to take a long walk without inconvenience. Next day reported himself entirely *cured.*

